# A Just Standard: The Ethical Management of Incidental Findings in Brain Imaging Research

**DOI:** 10.1017/jme.2021.38

**Published:** 2021

**Authors:** Mackenzie Graham, Nina Hallowell, Julian Savulescu

**Keywords:** Incidental Findings, Brain Imaging, Ethics, Research Results, Distributive Justice

## Abstract

Neuroimaging research regularly yields “incidental findings”: observations of potential clinical significance in healthy volunteers or patients, but which are unrelated to the purpose or variables of the study.

Neuroimaging scans taken in the course of research regularly yield “incidental findings”: observations of potential clinical significance in healthy volunteers or patients, which are unrelated to the purpose or variables of the study. As the number of research studies using neuroimaging — particularly MRI (magnetic resonance imaging) but also CT (computed tomography), and PET-CT (positron emission tomography-computed tomography) — has grown, the sheer volume of scans being generated means that incidental findings have become increasingly common. How these findings should be dealt with is a source of continuing debate amongst neuroimaging researchers and bioethicists.^1^ Two related questions dominate the discussion: to what extent should neuroimaging researchers look for incidental findings, and what should be disclosed to participants when an incidental finding is discovered. Various arguments have been presented attempting to justify an obligation to look for incidental findings and to disclose them to participants, including the researcher’s ancillary care obligations, the participant’s right to control information about themselves, and more general concerns of beneficence and autonomy. Conversely, opposition to disclosure has tended to focus on the burden this would place on researchers and the health system, and the risks of unnecessary harm that disclosure places on participants. While these positions have been well-articulated in the literature, little progress has been made in resolving this debate.

In this paper, we take a different approach to addressing the problem of how best to manage incidental findings. Rather than focussing on whether disclosure is or is not consistent with the interests of participants, we consider what participants are owed as a matter of distributive justice. While considerations of autonomy and beneficence are certainly relevant to this discussion, they offer only limited practical guidance. Essentially, beneficence requires that researchers look for incidental findings — and subsequently disclose them — when doing so is likely to be beneficial to the participant, and the costs are easily bearable relative to the potential benefit. For some incidental findings, it is relatively straightforward to determine when this requirement is met (e.g., when a researcher is highly confident that the finding represents a brain tumor). But in many cases, the clinical significance of an incidental finding is unknown.^2^ It will not always be obvious whether disclosure will provide a benefit, and thus, whether looking for incidental findings is likely to be beneficial. Similarly, not all instances of disclosure will promote participant autonomy, and identifying these cases can be difficult.

We argue that researchers have an obligation to look for and disclose incidental findings to participants only insofar as doing so is required by distributive justice. As citizens, research participants are entitled to a certain level of basic care, which the state has an obligation to provide. Researchers working in publicly funded institutions, or whose research is funded at least in part by the state, must carry out their research in a way that is consistent with this obligation, and not deprive research participants of the care to which they would normally be entitled outside the context of research. Accordingly, this paper will focus primarily on neuroimaging research being conducted in institutions that are at least partially publicly funded, within a broader health-care context in which imaging is available. As we will show, looking for incidental findings — and subsequently disclosing them — is in many cases not required to meet this obligation of basic care. In these cases, researchers are not obligated to provide them. Appealing to considerations of distributive justice thus has an advantage over beneficence and autonomy in that determining the requirements of justice does not require making a potentially under-informed judgement about whether a participant will or will not be benefitted (or have their autonomy pro-moted) through disclosure.

## Incidental Findings

Incidental findings are not uncommon in research imaging of the brain. Meta-analyses estimate that the overall prevalence of incidental findings in brain imaging ranges from 2.7% to 22% of cases,^3^ with the likelihood increasing with age and the sensitivity of the scan. Individual studies have recorded incidental findings in as many as 32%-34% of participants.^4^ Overall, between 1.4% and 8% of detected incidental findings require immediate referral for clinical evaluation, between 1.8% to 43% require routine referral, and between 13% and 40% require no referral.^5^ In a neuroimaging context — which will be the focus of this paper — findings can range from the benign, (e.g., asymptomatic arachnoid cysts, perivascular spaces) to the potentially serious (e.g., brain tumor, aneurysm, or arteriovenous malformation). However, many incidental findings which do generate a referral are of uncertain or unknown significance. Royal and Peterson found that 17% of 641 participants had an incidental finding of unknown clinical significance, of which 3% received a routine referral and 1% an urgent referral.^6^ There is limited data on what proportion of these incidental findings do in fact receive follow-up, and on the outcome for patients, particularly in brain imaging studies.^7^ One study by Shoemaker and colleagues found that 63% of participants who were recommended to see a physician did so, and of those participants, 38% received additional medical testing.^8^

Recommendations for the feedback of incidental findings have been published by various sources.^9^ These recommendations emphasize the importance of a clearly articulated plan for the feedback of incidental findings, and important considerations in its development (e.g., potential harms and benefits to participants, effective communication strategies). However, the content of the plan is typically left open to researchers, meaning that the practice of disclosing incidental findings can vary considerably. Generally, management of incidental findings falls into one of four categories: 1) no review (scans are not reviewed for incidental findings, and no information is conveyed to the participant; 2) select review (MRI technologist or radiographer ‘flags’ suspicious findings for further review by a radiologist, with limited findings returned to participant; 3) full review (all scans are analyzed by a radiologist, and all findings are reported to participants; 4) clinical scan (some research centres, including the US National Institute of Health, conduct a complete clinical scan in addition to the research scan, which is reviewed by a radiologist and all findings disclosed to the participant).^10^

In contrast to the diversity that exists in practice, there is a rough consensus amongst bioethicists that at least some incidental neuroimaging findings ought to be disclosed to participants, specifically, when doing so is consistent with their best medical interests. This consensus is based on the idea that researchers bear at least some care obligations to participants. On most accounts, three considerations are relevant to determining whether disclosure of an incidental finding is consistent with a patient’s best interests: scientific validity, clinical utility, and actionability. Scientific validity refers to the accuracy and reliability of the finding: was the scan conducted properly, and has an expert consultant confirmed the finding is suspicious, or could this be a measurement artifact? Clinical utility refers to the potential health or reproductive importance of the findings to the patient. This includes findings whose welfare implications are relatively immediate, or may be in the future (e.g., where the patient is a child and findings may only have an impact on their adult lives), as well as findings that might have implications for people other than the participant (e.g., the participant’s children or family members). An incidental finding revealing a brain tumor, arterio-venous malformation, or an equally life-threatening condition, would have clear clinical utility, whereas the discovery of a slight malformation of the amygdala lacks clinical utility, because there is no known significance to such a variation. Actionability refers to the potential for treatment or management of the underlying health condition revealed by the finding. An incidental finding revealing an unruptured aneurysm could lead to treatment through surgery, or the participant might choose to avoid playing contact sports. Conversely, nothing can be done in response to an incidental finding revealing slightly enlarged ventricles.

The combination of the uncertain nature of the findings, and their uncertain significance to the participant, means that considerations of autonomy do not clearly support the disclosure of a large proportion of incidental findings. Disclosing findings of clear clinical significance provides participants with information that they can use to better understand themselves or inform future decisions.

Most commentators agree that returning incidental findings which are actionable, scientifically valid, and have clear clinical significance to participants, is ethically justified. Of course, the obligation to disclose incidental findings must be balanced against the costs to researchers of doing so. Researchers may not be required to provide feedback of incidental findings if this would place a significant burden on their research (e.g., in terms of time, cost, or other resources). The primary source of disagreement within the literature concerns findings of uncertain or unknown clinical significance. Whether disclosing these findings provides a net benefit to participants, or demonstrates respect for their autonomy, is disputed. It is a further question whether disclosure is a justifiable use of research resources, or the resources of the broader health-care system in the event that referral is required. Moreover, these difficult cases highlight the lack of consensus about how to interpret the obligations of researchers to participants.

## Autonomy

The obligation to respect the autonomy of research participants has often been used as a justification for making incidental findings available to them. National and international regulations articulate the right of individuals to choose whether they wish to be informed of the results of genetic examination, for example, including a right not to be informed.^11^ The basic idea is that researchers ought to respect the capacity of participants to make their own decisions (i.e., to “self-determine”), and that the disclosure of incidental findings can have a significant impact on their capacity for self-determination. In discussing the disclosure of incidental findings in genetics research, Roberto Andorno argues that participants should be free to make their own choices with respect to medical information, rather than have this choice imposed on them by a third party (i.e., the researcher).^12^ Similarly, considerations of autonomy could be used to justify an obligation to look for incidental findings. Failing to look deprives the participant of potential information about themselves which might inform their future decision-making, and takes decision-making power out of their hands by restricting the possible information that a researcher has available to disclose.

The importance of autonomy emerges from the value that individuals place on acting according to considerations that they genuinely feel are their own, or that they view as aspects of their authentic self. Thus, a key component of exercising autonomy is a knowledge and understanding of oneself, of the things that one genuinely desires or values, and the ability to competently act on these desires and values. Information from incidental findings might be of consequence to a person’s future decision-making, either by directly informing a health decision, or by influencing self-knowledge (e.g., I may be less likely to pursue certain activities if I know I have a brain aneurysm). Accordingly, not knowing this information can frustrate autonomy, by inhibiting a person’s ability to make a fully informed decision. Having more information about themselves and their circumstances allows a person to take a more complete range of factors into account, predict the future more accurately, and make decisions that are in line with their values.

The critical role of information in promoting autonomy supports an obligation on the part of researchers to make incidental findings available to participants. In arguing for the obligation of researchers to return individual research results to participants, David Shalowitz and Frank Miller point out that showing appropriate respect for a participant’s contributions to research requires not treating them as a ‘mere means’ to accomplishing research goals.^13^ They argue that researchers ought to give due consideration to a participant’s interest in receiving information about themselves derived from their participation in research. The same rationale could be used to justify making incidental findings available, insofar as these findings might reveal information that would enhance a participant’s ability to self-determine.

Similarly, Judy Illes and colleagues argue for a reci-procity-based obligation on the part of researchers to make incidental findings available to participants.^14^ Participants contribute to the research enterprise by helping to produce generalizable health information, and researchers ought to reciprocate this benefit in kind, by providing participants with information that might be useful to them. Conversely, failure to disclose incidental findings that would be useful or valuable to the participant might be seen as actively inhibiting a participant’s capacity to self-determine, by removing or inhibiting their decision-making power (i.e., by not disclosing an incidental finding, the decision of how to respond to this information is taken out of the hands of the participant).

The close relationship between information and autonomy accounts for why many kinds of incidental findings might *not* promote participant autonomy if disclosed. If a participant would greatly misunderstand the implications of an incidental finding, it is not clear that disclosure would enhance their autonomy. Suppose a participant were informed that they had a brain cyst which was likely benign, but further examination would be required to confirm this. If the patient mistakenly believed that this cyst was likely to be fatal, and consequently sold off all their possessions, we would not say that their autonomy was enhanced by disclosure. Autonomous self-governance can hardly be promoted by an inaccurate or distorted understanding of information about oneself. In this example, the researcher disclosing the finding would need to take steps to ensure that the participant understood the information being presented.

However, in cases where the incidental finding itself is of uncertain or unknown significance, it is difficult to see how this provides meaningful information that can better reveal the nature of future options. Put another way, if an incidental finding is not informative in some way to the participant, then disclosing it does not enhance their ability to self-determine, and may in some cases detract from it (i.e., if it leads to unjustified conclusions about their health status). The very nature of neuroimaging in a research context means that many incidental findings will be of uncertain or unknown significance.

First, the quality of the image produced by research scans, compared to clinical-grade scans, makes it difficult to distinguish between pathology, benign anomalies, and artifacts in the scan itself. Most imaging research uses T1-weighted images. These scans can detect anatomical distortions associated with large, space-occupying lesions, but not more subtle anomalies, like evidence of tissue edema or necrosis. On a T1-weighted scan, the presence of a tumor would appear as a distortion of the anatomical structures surrounding the tumor; the tumor itself cannot be seen. In fact, a neurological examination or a thorough review of possible symptoms is likely to be more sensitive and specific in detecting clinically significant, symptomatic lesions than a research quality scan.^15^ A T2-weighted image, by contrast, is more clinically useful (though still less than a clinical-grade scan) but much less commonly used in research. Even when using a T2-weighted image, clinically relevant anomalies can go undetected, or appear as ‘unidenti-fied bright objects’ on the scan.^16^ Echoplanar images, a research-quality scan used mostly for functional neuroimaging, offers extremely poor resolution and is highly prone to artifacts, making them much less useful for the purposes of gleaning significant incidental findings.^17^

Second, even when an incidental finding can be clearly identified, the significance of the finding may remain uncertain. Many incidental findings are asymptomatic abnormalities which could have gone undetected and untreated for the duration of the participant’s life, if not for a research scan. Because these abnormalities usually remain undetected in the general population, little information is available about their natural history. This makes predicting their outcome upon detection as an incidental finding uncertain. For example, Chiari I malformations are an incidental finding detected in approximately 3% of healthy volunteers, but the relationship between the malformation and symptoms is uncertain.^18^ Nevertheless, the detection of such an anomaly can sometimes lead to a recommendation to avoid certain physical activities such as contact sports, or warnings about possibly increased susceptibility to neck trauma, as can occur in a motor vehicle accident. While disclosing such a finding to a participant conveys to them a new piece of data about themselves (they have a kind of brain malformation called a Chiari I malformation), it is not clear that this data is informative. A recommendation to avoid contact sports suggests that the participant is at some elevated risk, but the precise risk is unknown (indeed, it could be near-zero). It is easy to see how this data could lead a participant to make unjustified inferences about their health, or precipitate potentially unnecessary lifestyle changes that could have far-reaching consequences.

The combination of the uncertain nature of the findings, and their uncertain significance to the participant, means that considerations of autonomy do not clearly support the disclosure of a large proportion of incidental findings. Disclosing findings of clear clinical significance provides participants with information that they can use to better understand themselves or inform future decisions. Similarly, disclosing incidental findings of clear non-clinical significance (e.g., misattributed paternity based on a genomic sequence, often cited as a paradigm case of a significant incidental finding unrelated to health^19^ ) can promote participant autonomy. When findings are of unknown or uncertain significance, disclosure does not necessarily promote patient autonomy, and may in fact impede it. Indeed, empirical research suggests that participant understanding of incidental finding reports is mixed,^20^ meaning it may be difficult to predict whether disclosing certain kinds of findings will promote participant autonomy. For these sorts of cases, respect for participant autonomy does not provide a useful justification for disclosure.

## Participant Interests

Perhaps the most common justification of a researcher’s obligation to disclose incidental findings to participants is beneficence. The basic idea is that researchers have certain obligations to promote the well-being of participants, and that insofar as disclosing incidental findings can be expected to benefit the recipient, researchers ought to make these results available.

For example, Susan Wolf and colleagues^21^ propose a three-tiered system of disclosure, based on the potential net benefit to the participant. Incidental findings with “strong net benefit” are likely to offer much more benefit than burden to the participant, and ought to be disclosed. These include life-threatening conditions like brain tumors. Findings with “possible net benefit” may offer more benefit than burden and include findings that reveal a serious but untreatable condition, but that the participant would likely deem important. Researchers are not obligated to disclose these findings but may choose to make them available. Findings with “unlikely net benefit” should not be offered to participants, because disclosure likely offers more burden than benefit. These include findings not likely to be of serious medical importance, or whose importance cannot be ascertained, such as cysts, enlarged ventricles, or Chiari I malformations.

There are several potential sources for a researcher’s obligations to promote the well-being of research participants. First, it is widely accepted that we have general obligations to promote the well-being of others, simply in virtue of being moral agents; there are certain things we owe another individual who needs help, independently of any relationship we might have to them. One such duty is that of easy rescue. T.M. Scanlon articulates the duty thusly: “…if you are presented with a situation in which you can prevent something very bad from happening, or alleviate someone’s dire plight, by making only a slight (or even moderate) sacrifice, then it would be wrong not to do so”.^22^ Peter Singer makes a similar claim: “If it is in our power to prevent something bad from happening, without thereby sacrificing anything of comparable moral importance, we ought, morally, to do it”.^23^

Numerous commentators have appealed to the duty of easy rescue to justify a researcher’s obligation to actively look for incidental findings, and to disclose incidental findings to participants when they are discovered. Insofar as the cost to the researcher of disclosing an incidental finding is not overly burdensome, and the potential benefit (or harm avoided) to the participant is large relative to the cost, disclosure constitutes an “easy rescue,” and the researcher ought to do it. The same considerations justify a duty to look for incidental findings: if looking for an incidental finding is not overly burdensome, and the potential benefit (or harm avoided) to the participant is large relative to the cost, researchers ought to do it. Just as the general duty to promote the well-being of others obligates researchers to perform easy rescues through disclosing incidental findings, this general duty obligates them to bring about circumstances where easy rescues can be performed by looking for incidental findings.

The duty of easy rescue clearly justifies an obligation to disclose the most serious incidental findings, such as life-threatening brain tumors, about which there is already wide-spread agreement. However, it is less obvious that the duty of easy rescue generates an obligation to disclose incidental findings with low clinical significance, or of unknown clinical significance. Indeed, whether disclosure is likely to benefit the participant, and moreover, whether this benefit is outweighed by the potential harms of disclosure, is often uncertain. Arguing that researchers ought to disclose those findings which constitute easy rescues provides a useful justification for the obvious cases but fails to address the difficult cases which are most problematic for researchers.

A similar weakness applies to the justification to look for incidental findings based on the duty of easy rescue. It is often unclear when looking for incidental findings can be expected to benefit participants. In cases where research participants are healthy volunteers, with no symptoms to indicate a potential brain abnormality, researchers would have no reason to expect that a given participant might have a serious abnormality, and thus, might be benefitted by looking for an incidental finding (and subsequent disclosure). The potential benefit for any given participant would be based on the prevalence of serious asymptomatic brain abnormalities in the general population. Conversely, if the participants were not healthy volunteers but patients with health conditions known to the researchers, or were a population known to be at increased likelihood of potentially serious incidental findings (e.g., an elderly population) this might increase the likelihood of an incidental finding which the participant would benefit from having disclosed, and thereby provide a stronger justification to look.^24^ However, estimates of the prevalence of brain abnormalities across different populations are based on *detected* abnormalities, and so would represent only a lower boundary of the true rate of incidental findings in that population.

In fact, it is not clear that looking for potentially significant incidental findings benefits participants overall. Researchers from the UK Biobank compared two strategies for detecting incidental findings in a sample of 1000 healthy participants: systematic review of all research scans by a radiologist, and radiographer “flagging” of potentially serious incidental findings for subsequent review by a radiologist.^25^ Systematic radiologist review generated incidental findings in 179 participants, with subsequent clinical follow-up yielding a diagnosis considered serious (e.g., a tumor) in 21 participants. Conversely, radiographers flagged 66 participants, of which 18 were confirmed by a radiologist to have a potentially serious incidental finding. Of these 18 participants, 5 received a diagnosis considered serious after clinical follow-up.

Compared to systematic radiologist review, radiographer flagging missed 16 of the 21 incidental findings later confirmed as serious but resulted in far fewer false positives (13 compared to 158). Nearly all participants with an incidental finding had a subsequent clinical assessment, resulting in large numbers of investigations, referrals, and procedures which ultimately proved unnecessary. Many participants reported a negative impact on emotional well-being, insurance and finances, and work or activities of daily living, predominantly those who ultimately received a non-serious diagnosis. It is also worth pointing out that even when a patient ultimately receives a serious diagnosis (i.e., a true positive) may not benefit from the disclosure of the finding. As discussed above, many incidental findings are asymptomatic, and would have remained undetected, meaning that much is unknown about their natural course. This makes it difficult to balance the risks and potential benefits of treatment, or non-treatment. For example, endovascular coiling and surgical clipping — two standard treatments for unruptured aneurysms — have an adverse outcome rate (including death) of 8.8% and 17.8% respectively.

It is often argued that disclosing incidental findings can cause unnecessary anxiety to participants, although some research suggests that this worry may be overstated.^26^ Moreover, depending on the resources available, the financial cost of radiologist review of neuroimaging scans may not be overly burdensome; one study estimates a cost of around $25 USD per scan.^27^ Some research funders provide support for the costs of disclosing incidental findings, but this is not yet universal. However, many research environments simply do not have access to ancillary support like neuroradiology review, suggesting that availability may pose a greater problem than affordability in some cases.

More importantly, finding and disclosing incidental findings is only the first step in managing them. Once it has been decided by a participant’s primary care physician to follow up on an incidental finding, this can trigger a “cascade effect,” where a chain of testing and re-testing is initiated.^28^ This can result in a significant financial cost which must either be borne by a public health system, or by the participant. A physician may order follow-up imaging not because they think this will lead to a better outcome, but because of inexperience, fear of liability, to appease a patient, or lingering uncertainty.^29^ As the number of research studies using neuroimaging continues to increase, the pressure on public systems to support the management of incidental findings may become untenable.

Conversely, many participants whose radiology reports recommend follow-up do not receive it. This can be because the participant’s primary care physician is unsure of how to proceed, because they are skeptical of the radiologist’s recommendations, or to avoid the aforementioned cascade effect.^30^ It may be beyond the capacity (or responsibility) of the researcher to ensure that participants receive the recommended follow-up. However, the inconsistency of follow-up in primary care further complicates a researcher’s assessment of how likely a participant is to benefit from disclosure.

A further worry is that focussing on clinical significance construes participant benefit too narrowly. Participants may derive non-medical benefit from the disclosure of an incidental finding, and thus, net benefit should be understood broadly, to incorporate concerns which are not strictly medical.^31^ For example, misattributed paternity is an often-cited example of an incidental finding in genomic research which may be important to participants without having any medical significance.

While a researcher may be better positioned to assess the clinical benefits of an incidental finding than a participant, the more broadly we understand the benefits (and harms) of an incidental finding, the larger the role of the participant in assessing the value of an incidental finding. Therefore, beneficence could be argued to require that researchers make available any incidental finding which could be of value to the participant, and allow them to determine for themselves if disclosure is beneficial.^32^

Yet incidental findings of unknown or uncertain clinical significance in a neuroimaging context are different than often-cited cases of valuable non-clinical information, like misattributed paternity, or the APOe4 genetic variant that is associated with Alzheimer’s Disease. Even if these findings have no clear implications for the participant’s health, they contain information that could promote or frustrate the participant’s interests in other ways. Mistaken paternity might provide clarity about one’s identity or lead to family turmoil. An increased risk of Alzheimer’s might compel someone to make care plans or lead to anxiety or depression. In any case, it is the participant who is best able to judge whether knowing this information is likely to benefit or harm them, because the participant will have more insight into their non-clinical interests than the researcher.

Conversely, many incidental findings of unknown or uncertain clinical significance in a neuroimaging context do not contain enough information for the participant (or researcher) to make a judgement about whether disclosure is in the participant’s best interests. Without clear implications to the incidental finding, there is seemingly no basis on which a participant can determine whether disclosure is on balance beneficial or harmful.

## Ancillary Care

While a strong argument can be made for a researcher’s duty to rescue when the diagnosis and intervention options are clear, we have seen that this is often not the case. Thus, rather than appealing to a researcher’s general beneficence obligations *qua* moral agent, we might instead think that researchers have certain additional obligations to participants in virtue of their relationship to them. For example, Henry Richardson and Leah Belsky argue that in addition to meeting the requirements of scientific validity, safety, keeping promises, or rectifying injuries, (and the general duty of rescue that applies to any moral agent) researchers also have certain ancillary-care obligations to participants.^32^ On their view, the relationship between researcher and participant is one of partial entrust-ment. Research participants grant limited discretionary powers to researchers when they consent to participate in a trial, and in doing so make themselves vulnerable to researchers. For example, participants authorize researchers to collect confidential medical information, to collect bodily samples or otherwise compromise their bodily integrity, or undertake medical procedures on them. Entrusting these discretionary powers creates certain responsibilities in the researcher, including providing ancillary care if study information reveals a need.

On their view, the scope of a researcher’s ancillary care responsibilities depends on the range of permissions granted to the researcher (i.e., on the extent of their discretionary powers), while the strength of the responsibility depends on the degree of subject vulnerability, and what is reasonable for the researcher to provide (e.g., given the constraints of their research budget, personnel time, effect on quality of research data). Researchers must exercise their discretionary powers with compassion, engagement, and gratitude. Finally, ancillary care obligations may be overridden by other factors, including limitations on financial and human resources.

Incidental findings clearly fall within the scope of a researcher’s ancillary care obligations. Participants waive an aspect of their right to privacy by allowing researchers to acquire images of their brains, and researchers accrue discretionary powers over how to deal with this information (e.g., whether to analyze it for incidental findings).

The vulnerability of the participant (i.e., how will the subject’s health be affected by the researcher’s exercise of their discretionary powers) depends on the type of finding. Some incidental findings (e.g., arteriovenous malformation, hydroencephalus, tumors) have high clinical significance, and their discovery makes the participant highly vulnerable to the discretion of the researcher; the researcher’s actions could have a significant impact on the patient’s health and well-being. Conversely, in cases where incidental findings have low or no clinical significance (e.g., sinusitis, mastoid fluid, cysts) the participant is not particularly vulnerable. How the researcher exercises their discretionary power in responding to the finding will not have a significant impact on the participant’s health and well-being.

Whether a researcher’s ancillary care obligations require disclosing an incidental finding thus depends on the type of finding, mediated by the quality of the scan. When a researcher detects an incidental finding that appears to be of high clinical significance, and the evidence is clear and reliable, (e.g., an arteriovenous malformation on a T2 scan) the disclosure requirement is strong. Conversely, when the finding appears to be of low or no clinical significance, and the evidence for this is clear and reliable, the disclosure requirement is weak. Here, the obligation to disclose might be easily outweighed by countervailing considerations (e.g., cost, potential harms to the participant).

Deferring to researcher judgement may be appropriate in individual cases, but it is not an appropriate standard for determining a general policy of disclosure for assessment by a research ethics committee, or to be conveyed to a participant during the informed consent stage.

When an incidental finding is of a type with unclear or uncertain clinical significance, or the evidence for this finding is uncertain, the ancillary care responsibilities of the researcher become less clear. Suppose a researcher (or a radiologist tasked with analyzing the scan) detects what they think is an incidental finding of low or uncertain clinical significance (e.g. a Chiari I malformation) on a T1 scan. Because of the low resolution of the scan, they recognize their initial assessment could be incorrect; the incidental finding might be something else, and potentially more or less serious. One could make a reasonable argument that the researcher ought to exercise her discretionary powers in a cautious way and refer the participant for further follow-up. However, a researcher who determined that the statistical likelihood of a clinically significant incidental finding was low, was genuinely worried about causing the patient unnecessary anxiety, and so opted against disclosure, would not clearly be acting contrary to her ancillary care obligations on Richardson and Belsky’s view.

Furthermore, the ancillary-care model does not account for the varying capacities of researchers to recognize incidental findings. For example, a significant proportion of current health research is “data-driven” (e.g., the development of machine-learning algorithms), in which researchers are outside of a care context. What sorts of care obligations would a data scientist using brain MRI scans to train a machine-learning algorithm have to the contributors of this data? It is highly unlikely that a data scientist would have the necessary expertise to identify potential abnormalities in a scan, nor would it be advisable to require these researchers to make a judgement about the clinical significance of potential abnormalities they did happen to detect.

Entrusting researchers to exercise their judgement regarding whether care is required will likely lead to different outcomes in borderline cases. However, the repeated calls for further ethical guidance^33^ suggests that many researchers may not be comfortable exercising their judgement in these cases. This is especially likely for more junior researchers, who may feel they lack the experience to exercise their discretion in this way. Further, the potential variation between research centres may make it difficult for participants to understand the kinds of results that will or will not be disclosed, given that this will depend in part on the judgement of the researcher and cannot be articulated in advance. Deferring to researcher judgement may be appropriate in individual cases, but it is not an appropriate standard for determining a general policy of disclosure for assessment by a research ethics committee, or to be conveyed to a participant during the informed consent stage.

## The Requirements of Distributive Justice

So far, we have argued that the management of incidental findings presents a major challenge to researchers. Disclosure practices are inconsistent, and attempts to justify the disclosure (or non-disclosure) of incidental findings by appealing to considerations of autonomy or beneficence have not led to much progress. The problem is that in many cases, the requirements of beneficence and autonomy are indeterminate. In the next section, we offer an alternative approach. Rather than asking what moral obligations researchers have to participants, we focus on what research participants are owed as a matter of distributive justice.

Rebecca Kukla suggests that the fundamental intuition that underwrites modern research ethics is that “one should never let the ends of scientific investigation interrupt or trump one’s basic moral treatment of those whose bodies are used in their pursuit.”^34^ Research involving human participants essentially involves using them as a means to some scientific end. Research ethics helps us to avoid using participants as a *mere* means. Whatever research participants are owed as members of society, this cannot be compromised for the purposes of research. One of the consequences of this intuition is that researchers must not knowingly prevent participants from receiving care that they are entitled to as a matter of distributive justice.

We take it to be uncontroversial that states have a duty of distributive justice to provide their citizens with access to basic healthcare. Roughly, basic care encompasses the kinds of interventions and services that citizens require to pursue their own conception of well-being, including access to personal medical services and preventive care, the provision of public health measures like vaccinations, and enforcement of safety standards.^35^ The duty of the state to provide access to basic care also encompasses the conduct of clinical research in the public interest.

While numerous commentators have argued that citizens are entitled to basic care as a matter of distributive justice, Douglas Mackay argues that researchers have a duty to provide it.^36^ In addition to any obligations that researchers have in virtue of their role as professionals or moral agents, they also have what Mackay terms ‘institutional obligations’. Researchers who are employed or have their research sponsored by agencies or departments that are funded by the state, have an institutional obligation to “conduct their research in a way that that is consistent with the state’s duty of distributive justice to provide its citizens with access to basic health care”.^37^ This is because the state may not authorize its agents to act in ways which are inconsistent with its obligations. Accordingly, researchers must provide participants with the best treatment to which they are entitled, or when offering an experimental treatment, there must be genuine uncertainty regarding which treatment is superior (‘equipoise’). For example, as Mackay argues, it would be impermissible for a researcher to offer participation in a trial in which the control arm would receive less than the basic standard of care which the state is obligated to provide.

As Mackay points out, the obligation of researchers to provide participants with either the best treatment that the state owes them (or in cases where they reject this, the best treatment they will consent to and are entitled to), or a treatment in equipoise with or known to be superior to it, applies under ideal conditions — that is, conditions under which the state does in fact provide its citizens with the care they are entitled to. In non-ideal conditions where the state cannot (or does not) provide its citizens with the treatment to which they are entitled, such as may obtain in developing countries, researchers must provide participants with the best treatment they are legitimately able to provide. For the purposes of this discussion, we will assume that most research involving neuroimaging occurs in more-or-less ideal conditions.

What constitutes the basic standard of care a state must provide to its citizens will depend on several factors, including the medical, economic, and cultural conditions of the state. A developing country with limited resources to divide between education, health care, infrastructure and so forth, will offer a different level of basic care than a developed country, even if the developing country uses its resources in a maximally just and efficient way. Thus, whether a treatment or intervention ought to be a part of basic care will depend on a combination of cost, the efficacy of the treatment, potential harms (e.g., side effects), accessibility and ease of delivery, local support systems, community values, and many other factors.

An important aspect of the state’s provision of basic care is the funding of research, to evaluate the efficacy of novel treatment interventions or procedures, and to assess existing systems of health care delivery. Essentially, research provides evidence to determine whether something should be a part of standard care, by systematically evaluating the above factors. Researchers must balance their obligations to individual participants with their obligations to wider society, who stand to benefit from the generation of scientifically valid data. This has important implications for the disclosure of incidental findings.

Are research participants entitled to have their incidental findings disclosed, as a matter of basic care? Further, are researchers obligated to look for and disclose these findings, given their institutional obligations? Healthy asymptomatic participants would not receive a neuroimaging scan outside the context of a research study. Neuroimaging to screen for brain abnormalities is not currently a requirement of basic care in most developed countries. It could be the case that routine brain screening becomes a part of basic care to which all citizens are entitled, if it were determined that this was an effective way of preventing disease that was cost-effective and could be made widely accessible. In this case, we might think that it ought to be provided as a matter of basic care. Indeed, genomic testing for the purposes of screening for genetic disease is becoming a part of routine care, though only for people with certain conditions (or relatives with those conditions). However, as the above discussion has hopefully made clear, there are good reasons against routine neuroimaging to screen for abnormalities. While a small minority of patients might benefit from having a serious abnormality detected earlier than it otherwise would have been, the risk of false positive findings, the lack of clear justification for treatment in most cases, the significant cost, and the existence of arguably more effective methods of detecting brain abnormalities (e.g., routine neurological exam), justifies keeping brain imaging in healthy asymptomatic people outside the scope of basic care.

Thus, if neuroimaging is not a part of the basic care to which healthy asymptomatic citizens are normally entitled, researchers are not required to generate diagnostic quality scans in addition to those necessary for research, to better detect incidental findings. Healthy asymptomatic people are not entitled to the kind of information which can be provided by a research-quality neuroimaging scan as a matter of basic care, and thus, researchers do not deprive them of anything to which they are entitled by failing to actively seek out this information. While one might argue that researchers are thereby failing to provide participants with a potential benefit, it is not a benefit to which they are entitled as a matter of basic care (setting aside the fact that in many cases, disclosure will not actually be a benefit).

Further, generating diagnostic quality scans, or devoting time and resources to actively seeking incidental findings in research-quality scans (e.g., sending them for expert analysis) presents a potential financial burden for researchers, depending on the number of participants, and the funding available for the study. If researchers must allocate resources for the management of incidental findings, and as a result are able to scan fewer participants, for example, this creates an opportunity cost. Additionally, an increase in participants being referred to primary care for further assessment of incidental findings places a significant burden on the wider health system, and potentially deprives other patients who are entitled to care. As discussed above, researchers have justice-based obligations to society, in addition to their obligations to participants. Thus, actively looking for incidental findings is only justified if it is consistent with a researcher’s justice-based obligations to the individual participant, and to society.

With respect to disclosing incidental findings to participants, we argue that researchers have an obligation to disclose clinically significant findings. Informing a participant of information with clear significance to their health is a requirement of basic care, because this information is necessary to allow participants to pursue the treatment they may require, and to which they are entitled. For example, if the standard of care for white matter lesions in an asymptomatic individual is continued clinical monitoring, an incidental finding revealing the presence of white matter lesions should be disclosed. Here, the requirements of justice are consistent with the requirements of beneficence.

On the other hand, when the clinical significance of an incidental finding is unknown or uncertain, disclosure is not a requirement of basic care. The institutional obligations of researchers require that they conduct their research in a way that is consistent with the state’s duty to provide its citizens with access to basic care; the care necessary to allow them to pursue their own conception of well-being. The state does not have a duty to provide citizens with any health information that could potentially be of use to them. For example, routine monitoring of blood pressure, cholesterol, and so forth are aspects of basic care because the costs of doing so are such that they can be provided to all citizens, there are accurate and effective means of testing, and there is a reasonable expectation of benefit to the patient. Some forms of health-care monitoring are made available only to certain groups (e.g., people over a certain age, or with other comorbidities) as a component of basic care, for the same reasons. Conversely, some forms of health-care screening are controversial (e.g., breast cancer, colon cancer) because it is unclear if it is an effective method of reducing cancer rates. Some patients certainly do benefit from screening, but unless enough patients benefit (rela-tive to cost), screening is not a justifiable component of basic care within a resource limited system. Neuroimaging to screen for potential brain abnormalities clearly does not pass this threshold. Accordingly, participants are not denied something to which they were entitled if researchers do not disclose incidental findings of unknown or uncertain significance.

Our argument is not simply about the cost-effectiveness of feeding back incidental findings. Indeed, it could potentially be cost-effective in some cases to disclose incidental findings to participants, even if it would not be cost-effective to offer brain scanning as a form of health screening. Participants in a research study are a far smaller population, and because they are already receiving a scan for research purposes, the costs are reduced compared to population screening. As a practical matter, when concerned with incidental findings of uncertain or unknown significance, it will be difficult to determine whether feedback would provide adequate participant benefit for cost, precisely because the benefits of disclosure are unknown. Setting this issue aside, our argument is that researchers are obligated to ensure that participants are treated in a way that is consistent with the state’s obligations to ensure a basic standard of care. Thus, researchers are not obligated to provide whatever is cost effective within the context of the research project; they are obligated to ensure that the obligations of the state to its citizens is met.

Moreover, disclosing incidental findings of unknown or uncertain significance may impede the satisfaction of researchers’ institutional obligations, by precipitating the expenditure of significant health care resources for participant follow-up, with only a small chance of benefit. Not only might these resources have been more beneficial if used for other patients (resulting in an inequitable use of resources), but delays in access to imaging-based tests may also cause harm to other patients who need to access these tests. To return to the UK Biobank study mentioned above, the researchers estimated that systematic radiologist review would generate an additional 15,800 false positives over seven years.^38^ While UK Biobank is a comparably large imaging study, it represents only a fraction of the number of neuroimaging scans which occur each year. The fact that a participant *could* benefit from the disclosure of an incidental finding and subsequent follow-up testing does not necessarily entitle them to receive it. Neither patients nor research participants are entitled to the best care *possible*, because this standard would require physicians and researchers to violate the requirements of distributive justice.

It is worth noting that the institutional obligations of researchers are in addition to their natural and professional obligations. In addition to duties of easy rescue (as discussed above), they also must comply with the requirements of ethical research, including scientific validity, fair subject selection, a favorable risk-benefit ratio, informed consent, and independent institutional review. As part of the informed consent process, then, participants have the right to be informed of the researcher’s procedure for managing incidental findings, including the kinds of incidental findings which are of sufficient clinical significance to trigger a referral, whether the participant would like to receive these sorts of findings, and timely communication if something is detected.

A further consideration here is how the obligations of researchers might evolve over time. Specifically, a disclosure policy which was consistent with the requirements of standard care when it was adopted may not be at some future time. This is potentially problematic for long-term research studies. For example, when UK Biobank began recruiting participants in 2006, it proposed to follow participants for 30 years thereafter. When data was initially collected, participants were informed that they would not receive incidental findings arising from the use of their data in research. However, if imaging analysis were to drastically improve over the next decade (which, with the continued development of machine learning algorithms to analyze brain scans, is not inconceivable), it is possible that brain imaging could become a part of standard care for asymptomatic people. In this case, might UK Biobank need to change its disclosure policy? On the one hand, they would be failing to provide information which participants are entitled to. On the other hand, it might be highly burdensome for researchers to require that they re-contact participants and ask them to amend their informed consent to receive feedback of incidental findings. We tentatively suggest that because the obligations of researchers depend on the standard of care in the wider health system, a significant change in the standard of care would require a shift in disclosure policy. However, competing factors (e.g., the burdens to researchers, the expected benefits to participants of a shift in policy, the ubiquity of the change in the wider health system) would also need to be given due consideration.

One might also argue that the disclosure of an incidental finding is separate from the follow-up and possible treatment that a participant might receive as a result. Therefore, even if participants are not entitled to further testing resulting from an incidental finding of unknown or uncertain significance, they are still entitled to have the finding disclosed. It is certainly true that the resources required to disclose a finding are less than the resources required to disclose and provide follow-up. If the only considerations against disclosure were resource allocation, disclosure without providing follow-up might be justified. However, it would be impermissible to disclose an incidental finding of unknown or uncertain clinical significance to a participant, without the possibility of follow-up. We have argued above that incidental findings of uncertain or unknown clinical significance do not constitute useful information about a participant’s health, and revealing this data about the participant’s brain without also providing them a means of interpreting or understanding it impedes their autonomy, and risks causing serious anxiety or other harm. Thus, if an incidental finding is not of sufficient clinical significance that it merits follow-up as part of the requirements of basic care, it should not be disclosed.

## Conclusion

We have argued that researchers have an obligation to look for and disclose incidental findings arising from neuroimaging research only insofar as this is a requirement of the basic care to which participants are entitled as a matter of distributive justice. While attempts to justify disclosure on the basis of respect for participant autonomy or beneficence (arising either from the researcher as a moral agent, or in their role as a researcher) provide a reasonable justification for disclosing incidental findings in cases where there is clear clinical benefit, they inadequately address researcher’s responsibilities regarding findings of uncertain or unknown significance. Because a large proportion of incidental findings are of this kind, this is a significant weakness for the standard accounts, and is a major reason for the lack of practical progress in this debate.

Appealing to considerations of distributive justice provides an answer for these difficult cases. Data about a patient’s brain that may be generated by a neuroimaging scan in a research context is not something a healthy participant is entitled to as a matter of basic care. Accordingly, a researcher has no obligation to generate this information by performing additional scans (i.e., to “look” for incidental findings). Similarly, if a researcher discovers an incidental finding of unknown or uncertain clinical significance, they are not required to refer a participant for follow-up. Screening for brain abnormalities is not a requirement of basic care, and the burdens of follow-up on the health system (given the potential benefits) are inconsistent with distributive justice. This approach thus avoids the problem of trying to determine whether disclosure (is likely to) promote autonomy or benefit the patient. Rather, it requires researchers to ensure that participants are not deprived of anything to which they are entitled as a matter of distributive justice. This includes all of the protections to which participants in research are normally entitled, as well as the disclosure of clinically significant incidental findings.

Research participants in neuroimaging make an important contribution to the development of generalizable knowledge, and this is worthy of respect. However, we should not assume that providing them with information is always in their interests. The best way for researchers to respect participants and demonstrate appreciation for their contribution to research is by treating them as justice requires.

## Note

This research was supported by a Small Research Grant from the British Academy/Leverhulme Trust awarded to MG, and by a Wellcome Centre Grant (203132). MG’s research is also supported by the National Consortium of Intelligent Medical Imaging (NCIMI) (Innovate UK Grant 104688).
